# Community Stakeholders’ Perspectives on Recruiting Young Adolescents (Age 10–14) in Sexual Health Research

**DOI:** 10.3390/healthcare13141711

**Published:** 2025-07-16

**Authors:** Sadandaula Rose Muheriwa Matemba, Sarah Abboud, Rohan D. Jeremiah, Natasha Crooks, Danielle C. Alcena-Stiner, Lucia Yvone Collen, Chifundo Colleta Zimba, Christina Castellano, Alicia L. Evans, Dina Johnson, Tremain Harris, Natalie Marie LeBlanc

**Affiliations:** 1 College of Nursing, University of Illinois Chicago, 845 S Damen, Chicago, IL 60612, USA; abbouds@uic.edu (S.A.); rjerem@uic.edu (R.D.J.); ncrooks@uic.edu (N.C.); 2 School of Nursing, University of Rochester, 255 Crittenden Blvd, Rochester, NY 14620, USA; danielle_alcena@urmc.rochester.edu (D.C.A.-S.); natalie_leblanc@urmc.rochester.edu (N.M.L.); 3 Department of Community Health Nursing, Kamuzu University of Health Sciences, Lilongwe P/Bag 1, Malawi; lcollen@kuhes.ac.mw; 4 National Organization of Malawian Nurses and Midwives, Lilongwe P.O. Box 30393, Malawi; chifundozimba@nonm.mw; 5 School of Nursing, University of North Carolina at Chapel Hill, Chapel Hill, NC 27599, USA; 6 Nursing Faculty & Staff Directory, College of Nursing, University of Missouri, St. Louis, MO 63121, USA; ccastellano@umsl.edu; 7 LeGray Dynamic, Rochester, NY 14611, USA; legraydynamic@gmail.com; 8 Monroe County Family Coalition, Rochester, NY 14626, USA; dyj9218@rit.edu; 9 Department of Recreation & Human Services, City of Rochester, Rochester, NY 14604, USA; tremainharris00@gmail.com

**Keywords:** community stakeholders, perspectives, sexual health research, young adolescents

## Abstract

**Background/Objectives**: Sexual health research involving young adolescents remains scarce despite rising rates of early sexual debut, pregnancies, and sexually transmitted infections (STIs) in this population. We explored community stakeholders’ perspectives on engaging young adolescents in sexual health research in Western New York to inform strategies for engaging young adolescents in sexual health research. **Methods**: This qualitative descriptive study was conducted from April 2022 to June 2023. Seventeen community stakeholders, including health education teachers, youth counselors, and adolescent health providers, participated in semi-structured in-depth interviews. Data were analyzed using conventional content analysis, managed by MAXQDA 2020. The rigor and trustworthiness of the data were ensured through triangulation with observations, peer debriefing, team analysis, and respondent validation. **Results**: Participants were predominantly female (94.1%), 52.9% Black/African American, 41.2% White, and 5.9% Caucasian–Indian American, and aged 23–59 years. Four themes emerged: perspectives on conducting sexual health research with young adolescents, recruitment strategies, sexual health questions appropriate for young adolescents, and building readiness for participation in sexual health research. Participants reported the need for sexual health research with young adolescents and recommended building a trusting relationship and involving schools, parents, and trusted community organizations in the research process. Suggested research questions included those related to awareness of sex, STIs, available resources, experiences with sexual education, and desired support. The findings also revealed the need to initiate sexual health conversations early when children start asking questions, as a foundation for meaningful participation in sexual health research. **Conclusions**: The findings suggest that sexual health research with young adolescents is feasible and necessary, with implications for the design of developmentally appropriate sexual health research and interventions grounded in trust and community collaboration. Future research should explore the perspectives of caregivers and young adolescents to inform studies and programs that are attuned to young adolescents’ developmental needs.

## 1. Introduction

Young adolescents (ages 10–14) are at a critical stage of development, when foundational attitudes, values, and behaviors related to sexuality begin to form and when they are particularly vulnerable to the emergence of mental and behavioral health challenges [1,2,3]. With the earlier onset of puberty and rising rates of early sexual debut, sexually transmitted infections (STIs), and early pregnancies among young adolescents [2,4,5], there is an urgent need to prioritize sexual health research and support for this age group. Despite the increased recognition of the need to engage young adolescents in sexual health research and support their developing sexuality, this population remains significantly underrepresented in sexual health research, both in the United States (US) and globally [3,6]. Thus far, much of the existing sexual health research has centered on older adolescents and young adults, overlooking a critical window of opportunity to understand and address the needs of young adolescents [4,7]. However, evidence shows that some engage in sexual activities, experience unintended pregnancies and births, and are diagnosed with STIs, including HIV and human papillomavirus (HPV), before their 15th birthday [4,8,9]. Community stakeholders, including parents, educators, healthcare providers, and youth-serving professionals, play a pivotal role in shaping how and whether young adolescents are recruited into sexual health research. Their perspectives are essential in designing research that is ethically sound, developmentally appropriate, and grounded in community trust. However, regardless of their central role in facilitating research with young adolescents, there is a significant gap in the literature examining community stakeholders’ views on involving young adolescents in sexual health research. Without this knowledge, researchers risk designing studies and interventions that fail to resonate with the very communities that they aim to serve. Understanding community stakeholders’ perspectives is essential to developing inclusive, contextually relevant, and culturally responsive research strategies that can improve access, equity, and outcomes in adolescent sexual health.

The past two decades have registered a significant change in the way that the participation of young adolescents in research is viewed in the United States (US) and globally [10,11]. Since the late 1980s, several multidisciplinary research efforts have highlighted the importance of shifting the roles of children and young adolescents from human subjects or objects to active participants [12]. Modern initiatives highlight the need to reposition research as being “about” or “with” children and young adolescents, rather than “on” them [12,13]. Lewis et al. [14] proposed shifting the decision-making process in sexual health research to one where young adolescents’ contributions are encouraged and valued as standard practice, as this population can provide critical insights into issues affecting their age group [14]. Additionally, the Institute of Medicine of the National Academies [15] highlighted three areas that support research with young adolescents. First, well-designed and executed research with children and adolescents is needed to improve the health of future generations worldwide; second, children and adolescents should not be excluded from participation in research; and, third, there is a need for a robust system for the protection of child and adolescent research participants and to recognize and address unique ethical issues associated with research with this age group. Similarly, Article 12 of the Convention on the Rights of the Child makes provisions for young people to be heard, taken seriously, and be consulted in the formulation of legislation and policy related to issues and other problem areas affecting them. Article 12 further states that young people should be involved in the drafting, development, and implementation of youth-related plans and programs, including research [16].

Engaging young adolescents in sexual health research is essential as it can inform early interventions that can help to prevent adverse outcomes such as unintended pregnancies, STIs, and experiences of sexual coercion or violence. Targeting ages 10–14 for sexual health research is critical because this is a developmental stage where adolescents begin to experience puberty, explore gender expressions, become curious, and develop a deeper understanding of their bodies and sexuality [17,18]. Research during this stage can provide insights into their knowledge, attitudes, and behaviors regarding sexual health, which can inform age-appropriate education and interventions [19]. Additionally, research with young adolescents offers an opportunity to address misconceptions and provide accurate information early and can help to prevent risky behaviors, reduce the rates of STIs, and promote healthy sexual development [20]. Engaging this age group in research also allows for the identification of gaps in their knowledge and the development of resources that meet their specific needs.

While the literature highlights the benefits of engaging young adolescents in sexual health research, many factors limit their involvement. These include the use of 15 as a minimum age for participation in sexual health research, discomfort around discussing sexual health issues, challenges in obtaining IRB approvals, and difficulties in designing age-appropriate questions and survey instruments [17,21]. Recruitment and engagement challenges, ambiguities around young adolescents’ legal status, and concerns about their capacity to understand research questions make sexual health research with this age group particularly complex. As a result, researchers often rely on older adolescents or adults to infer the sexual health needs of young adolescents [22,23]. However, despite concerns about their developmental capacity, evidence suggests that young adolescents are capable of understanding research questions and processes and can grasp essential elements of research participation [24]. For example, in a Canadian study evaluating children’s comprehension of the risks and benefits associated with biomedical research participation from age six through adolescence and adulthood, the results showed that, when provided with standardized procedures and questionnaires, most participants, regardless of age, understood the basic purpose and procedures of the research and selected correct responses [25].

Various developmental theories also provide a foundation for understanding young adolescents’ ability to participate meaningfully in research. Erikson’s theory of psychosocial development [26] positions young adolescents within the “industry vs. inferiority” and emerging “identity vs. role confusion” stages, which are characterized by a growing need for autonomy, competence, and social connectedness. These developmental milestones support adolescents’ motivation to be seen as capable and their readiness to contribute meaningfully to research processes. Similarly, Piaget’s theory of cognitive development places young adolescents at the transition into the formal operational stage, where they begin to demonstrate abstract reasoning, hypothetical thinking, and perspective-taking cognitive abilities that are essential for informed research participation [27,28]. Complementing these developmental perspectives, Hart’s ladder of youth participation offers a widely used framework for understanding varying degrees of youth involvement in research and decision-making, including in research settings [29]. Hart emphasizes the importance of engaging children and adolescents in meaningful projects with adults, arguing that active participation promotes self-understanding, a sense of agency, and the skills necessary for future civic engagement. He cautions that it is unrealistic to expect young people to become responsible participating adult citizens without prior opportunities to practice these roles through guided inclusive experiences [29]. These frameworks suggest that, with appropriate methods and safeguards, young adolescents can actively and ethically contribute to research, both as partners and research subjects [30].

The parental consent requirement is reported to further limit young adolescents’ participation in sexual health research and can bias samples toward adolescents who are comfortable discussing sensitive issues with their parents [21]. Researchers argue that such a requirement, especially in studies on stigmatizing topics, may compromise scientific quality and limit evidence-based interventions for issues like HIV/STIs [10,31,32,33], as studies have shown that young adolescents possess decision-making abilities and, by age 14, they are comparable to adults and can understand research’s risks and benefits [31,32,34]. Findings from a targeted review of the international literature also suggest that research with young adolescents should be developmentally appropriate and context-sensitive and take a strengths-based approach, viewing young adolescents as active contributors and change agents [19]. Nevertheless, protecting the rights and welfare of children and young adolescents is the primary reason for requiring parental or guardian consent when young people participate in scientific research [35], in alignment with the International Ethical Guidelines for Health-Related Research Involving Humans [36]. According to the Declaration of Helsinki, when individuals lack legal capacity, informed consent must be obtained from their legal guardians in accordance with national laws [31,37]. The literature also consistently emphasizes the importance of informing parents and guardians about their children’s participation in research, highlighting this as a fundamental ethical requirement in studies involving minors [35,36,37]. Equally, ethical guidelines require that researchers take into account young adolescents’ developmental stages when explaining the study’s purpose, procedures, and potential outcomes and when assessing their ability to provide informed assent [10,30,37].

To promote research with young adolescents, studies show that incentives are important in encouraging participation. Crane and Broome [24] found that incentives were generally viewed positively, although concerns about undue influence were highlighted. To address this, Braun-Courville et al. [21] recommend offering financial compensation while clarifying that it is not tied to the extent of participation, thereby reducing the risk of coercion. Nevertheless, beyond monetary incentives, research also shows that adolescents are motivated by altruism, a desire to contribute to meaningful research, and an interest in learning about their health and development [38]. Many adolescents view participation in research as a way to stay informed about emerging issues that affect their lives and communities [21]. These intrinsic motivations highlight that adolescents are not solely driven by financial rewards and deserve to be given meaningful opportunities to participate in research. Engaging them as informed and willing contributors not only respects their agency but also enhances the relevance and impact of sexual health research.

To effectively conduct sexual health research with young adolescents, it is essential to understand how to engage them in such studies. Community stakeholders, such as parents, educators, healthcare providers, youth-serving professionals, and faith or community leaders, play a key role in shaping the local context for research involving young adolescents. Their perspectives and support are essential in ensuring that studies are ethically conducted, culturally responsive, and practically feasible [39]. Stakeholders often serve as gatekeepers and advocates for youth and can either facilitate or hinder research recruitment and participation. Understanding their insights is particularly important in communities where historical mistrust of research or concerns about discussing sexual health with youth may exist [40,41,42].

Despite the critical role of community stakeholders’ input in research, few studies have systematically examined community stakeholders’ perspectives on recruiting young adolescents for sexual health research in US contexts. Existing studies have primarily explored stakeholders’ views on sexual health education for young adolescents [43], ethical considerations in conducting research with young adolescents [14,24], perspectives on mental health stigma [44], and youth involvement in disease-focused community interventions, such as diabetes prevention [45]. However, there is a significant gap in research on community stakeholders’ perspectives regarding the inclusion of young adolescents in sexual health research. This gap limits the field’s ability to develop ethical, effective, and community-informed and developmentally appropriate sexual health research strategies for young adolescents. This study addresses this gap by exploring community stakeholders’ perspectives on recruiting young adolescents (ages 10–14) into sexual health research in Western New York. We pursued two aims: (1) to explore how stakeholders view the feasibility of interviewing 10 to 14 year olds about their experiences with sexual health support and (2) to identify best practices that reflect the social and cultural contexts of research with young adolescents through community engagement. By engaging with community stakeholders, we hoped to uncover strategies that would make sexual health research more inclusive, ethical, and responsive to the needs of young adolescents. The findings from this study can inform the development of ethical recruitment strategies for young adolescents in sexual health research and support the design of young adolescent-centered sexual health research in the US and beyond.

## 2. Materials and Methods

We used a community-based participatory research approach [46,47] that involved multiple phases. We first consulted organizations and schools that worked with young adolescents in Rochester, NY to determine the need for research and ensure that the study was relevant to the community. We worked closely with four community partners who served as community research consultants throughout the project. Together, we co-designed a four-phase qualitative descriptive study that was responsive to community needs and priorities. Phase one of the study involved key informant interviews with 17 community stakeholders, including providers at school-based health centers, health education teachers, and staff from youth-focused community organizations. This phase aimed to clarify the nature of sexual health development support for young adolescents, identify existing resources, and assess unmet needs and the feasibility of conducting research with young adolescents. Phase two focused on interviews with 20 parents and primary caregivers of 10–14-year-old adolescents to understand their experiences of supporting their children’s sexual development and their views on involving young adolescents in sexual health research. Phase three engaged 30 older adolescents (ages 15–19) through a youth advisory board (*n* = 14) and listening sessions (*n* = 16) to gather youth-informed perspectives on adolescent-focused sexual health research and to co-design a study that would include younger adolescents. Phase four, which has recently been approved by our IRB, was designed to engage young adolescents (ages 10–14) in individual interviews or focus group discussions to explore their understanding of sexual health; their experiences navigating puberty, forming and maintaining healthy relationships, and making decisions about their sexual health; their perspectives on sexual health education in schools; and the type of support that they need. This manuscript reports findings from phase one, which used a qualitative descriptive design [48] to explore community stakeholders’ experiences and perspectives related to sexual health development support for young adolescents. We selected this design given the limited existing research on sexual health development support for this age group.

### 2.1. The Conceptual Framework

**Figure 1:**
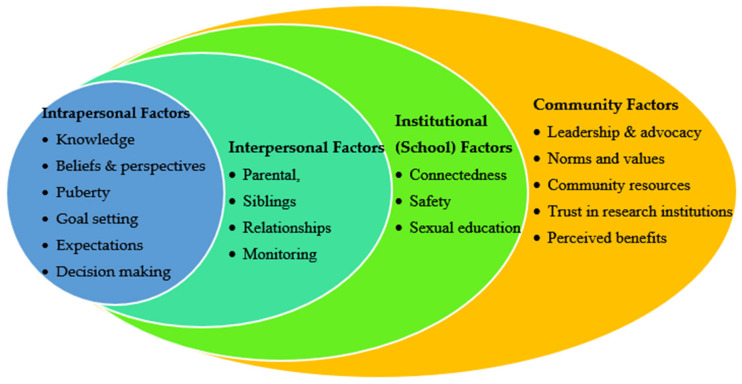
The socioecological model for health promotion as applied to young adolescents’ sexual health development support, adapted from McLeroy et al. [49].

This study used McLeroy et al.’s [49] socioecological model for health promotion as the guiding framework to explore community stakeholders’ perspectives on recruiting young adolescents into sexual health research. The model is particularly well suited to this inquiry as it conceptualizes health behavior as shaped by the dynamic interplay of multiple levels of influence: intrapersonal (individual), interpersonal (relationships), institutional, community, and public policy. Sexual health development support and young adolescents’ participation in sexual health research is similarly influenced by factors operating across these levels [50,51]. Our four-phase study focused on the first four levels of the model: community, institutional, interpersonal, and intrapersonal. In this phase, we began with the community level to examine the broader structural, cultural, and normative forces that shape whether and how young adolescents can be ethically and meaningfully engaged in research. Community-level factors such as prevailing sociocultural and religious norms, historical mistrust of research institutions, and local attitudes toward adolescent sexuality can either facilitate or hinder youth participation [39]. Research shows that, in communities where sexual health is stigmatized, youth voices are not prioritized, or there is a history of research exploitation, adolescent participation is often viewed with suspicion or resistance [39]. Conversely, in communities that actively support adolescent well-being and where youth-serving organizations are trusted, participation in sexual health research is more likely to be encouraged and supported [52,53,54]. By applying the socioecological model in a phased, layered manner, starting from the community, moving through interpersonal and institutional, and ending at the intrapersonal level, we aimed to capture the full complexity of ethical and practical considerations that influence the inclusion of young adolescents in sexual health research. Figure 1 illustrates how the socioecological model was applied in this study.

### 2.2. Sample and Sampling

We employed the maximum variation and snowball purposive sampling methods [55,56,57] to invite subjects from the community, school-based health centers, adolescent clinics, and schools to participate. We selected a range of sampling variation based on socioeconomic status, ethnicity, the age of the young adolescent, their grade, and the community organization’s institutional mandate or their role with young adolescents in order to capture diverse perspectives on conducting sexual health research with young adolescents. We conducted semi-structured in-depth interviews from April 2022 to June 2023. Our final sample size was 17, a point at which we reached saturation. The procedures and analyses of this study were approved by our institutional review board.

### 2.3. Recruitment

We used active and passive recruitment strategies. We circulated study flyers via email, posted flyers in healthcare facilities, and used personal connections with community organizations working with young adolescents. Subjects were asked to contact the study team directly and complete a screener form in REDCap to assess their eligibility. Eligibility included being over 18 years old, speaking and understanding English, and having a youth-related role in Monroe County, Rochester, NY. Participant screening was conducted by study staff through phone, Zoom, in person, and via an online screening survey created in REDCap. All eligible participants received an informational letter outlining the study details. After reviewing the letter and receiving further explanation from the study staff, participants were asked whether they wished to participate in the study. They were required to respond “yes” to proceed or “no” to decline. We obtained a waiver for written consent from the IRB as the consent process was primarily conducted remotely via phone or Zoom. For those screened through REDCap, after reading the information sheet, participants were prompted to indicate their interest by selecting either a “yes” or “no” checkbox. If they selected “yes”, the principal investigator was immediately notified and followed up with a phone call or email to schedule a one-on-one interview. Interested participants also provided their sociodemographic and contact information.

### 2.4. Data Collection

The interviews were conducted via Zoom and in person, based on the participant’s preference, and lasted 35–65 min. Each participant received USD 30 for their time. A semi-structured in-depth interview guide (see Supplementary Materials) was used to guide the conversation. The participants were asked the following domain questions: (1) How do you feel about having conversations with young adolescents to learn more about what support they are getting when it comes to puberty, relationships, and making decisions about their sexual health? (2) What are the effective strategies for engaging adolescents aged 10–14 in discussions about their sexual health in a research setting? (3) What specific questions should be asked to capture their experiences with the guidance and resources available to them? The interview guide was pilot-tested with two participants, with the senior author observing both interviews. A debriefing session followed each interview to identify any unclear or confusing questions, which were subsequently revised. These pilot interviews were excluded from the main analysis. To mitigate the risk of social desirability bias, we took several steps during data collection. First, the interviews were conducted in private settings by trained research staff who emphasized confidentiality and the voluntary, non-judgmental nature of participation. The interviewer also built a rapport with the participants and used open-ended questions, probes, and different forms of questioning to encourage authentic responses. Participants were reminded that there were no right or wrong answers and that their honest perspectives, positive or critical, were valued.

### 2.5. Data Analysis

The interviews were transcribed verbatim by a transcription company. Data analysis followed a conventional content analysis approach. This qualitative content analysis involved an inductive iterative approach in the generation of codes and categories and then themes and subthemes [58]. The first, second, and senior authors read each transcript repeatedly from beginning to end to achieve data immersion and obtain a sense of the whole, while identifying the underlying concepts and clusters of concepts [58,59]. These authors highlighted text, keywords, or phrases that appeared to describe perspectives on conducting sexual health research with young adolescents. They also made notes of their first impressions, thoughts, and initial analyses. As this process continued, codes were developed that reflected one or more critical thoughts that came directly from the text and the interpretation of the text. Coding continued systematically across the entire data set, with words, lines, and entire passages either forming a new code or fitting into an existing code [58,59,60]. Through this process, facilitated by the MAXQDA 2020 software, we developed our initial codebook.

After developing the initial codebook, our community research partners and other co-authors read three randomly selected transcripts and shared their interpretations of the results in a meeting. This process led to the development of our final codebook. After coding all transcripts, the researchers examined and discussed each code. This process led to combining some codes to form main categories and themes and splitting others into subthemes. Memoing was also applied during this process to deepen our analysis, generate new ideas, and interpret and communicate our findings. Memoing assisted in making conceptual leaps from our raw data to those abstractions that explained perspectives on sexual health research with young adolescents [61]. Next, we documented the definition of each theme and subtheme in the codebook, a process that helped in identifying important interpretations [57]. Peer debriefing was conducted throughout this process to ensure agreement with the coding process and definition of themes. The first author conducted member checks with the participants individually. She presented the findings and received feedback. To further ensure integrity in the analysis, we involved community partners throughout the study analysis process to help to shape the language and interpretation of the findings and to ensure that they were culturally responsive. We also engaged in an ongoing reflexive dialog, triangulated the findings among academic and community team members, and carefully interpreted the responses within the context of participants’ roles and expertise.

### 2.6. Positionality Statement

The authors of this manuscript comprised a multidisciplinary team with diverse academic and community-based expertise. The team included a professional community nurse, a community health nurse lecturer, assistant professors, an associate professor, and a professor, with backgrounds in adolescent sexual health, qualitative research, and community-based participatory research (CBPR). Our team also included community partners who worked directly with young adolescents through neighborhood-based programs and community organizations. These community partners brought not only professional experience but also lived connections to the communities that we aimed to understand. One partner held a Master’s degree in Leadership and Organizational Change and was a youth coordinator for the City of Rochester, responsible for the Mayor’s Youth Academy, the Mayor’s Youth Advisory Council, and the Biz Kid$ Entrepreneurial program with more than 27 years of experience in working with youth and leading community engagement initiatives. Another was a Bachelor-prepared serial entrepreneur, motivational speaker, and outreach specialist dedicated to empowering young people. A third partner was a community advocate with a Master’s degree in Nonprofit Management and Leadership, who served as the CEO and founder of a grassroots organization working with underserved youth and families, with a focus on substance use and health disparities. The fourth was a Masters-prepared structural strategist with expertise in communication, culture, and women and gender studies. These community partners were not recruited solely for this study; rather, their involvement emerged organically during the early stages of the project, through preliminary consultations and listening sessions with the community. Their insights and relationships helped to shape the direction of our work from the outset.

As academic and professional researchers, we understood ourselves as outsiders to the lived experiences and daily realities of the young adolescents who were the focus of this study. In contrast, our community partners, embedded in the neighborhoods that we studied, were the insiders. They held trusted relationships with the youth and their families and had firsthand knowledge of the cultural and structural dynamics shaping young adolescent sexual health development in these contexts. This insider–outsider collaboration was central to our approach. It allowed us to co-create a study that honored the voices and lived realities of young adolescents, while also grounding our methods in scientific rigor. The combination of academic knowledge and community-rooted expertise helped to ensure that the research questions, data collection tools, and interpretations were both contextually relevant and methodologically sound. Collectively, the team brought a depth of experience in addressing sexual health issues, engaging adolescents across diverse community and institutional settings, and applying qualitative and community participatory approaches to research.

To mitigate potential biases stemming from the researchers’ outsider status, we engaged in an ongoing reflexive dialog throughout the study, involved community partners in key phases of recruitment and interpretation, and triangulated the findings with input from both academic and community team members. This collaborative, iterative process ensured that multiple perspectives shaped the development of the research questions, coding frameworks, and interpretation of the findings.

## 3. Results

Table 1 shows the sociodemographic characteristics of the community stakeholders who participated in the study.

**Figure 2:**
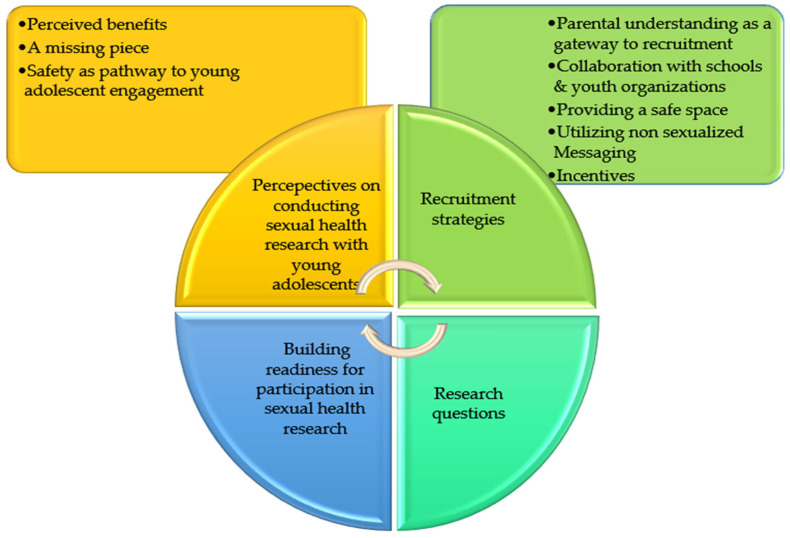
Themes emerging from the participants’ narratives.

The community stakeholders’ responses revealed the following four key themes: (1) perspectives on conducting sexual health research with young adolescents; (2) recruitment strategies; (3) research questions; and (4) building readiness for participation in sexual health research. Figure 2 shows the major themes and subthemes that emerged from the participants’ narratives.

### 3.1. Community Stakeholders’ Perspectives on Conducting Sexual Health Research with Young Adolescents

Under this theme, we identified the following three subthemes: (1) perceived benefits, (2) a missing piece, and (3) safety as a pathway to young adolescent engagement.

#### 3.1.1. Perceived Benefits

The findings showed that community stakeholders strongly supported conducting sexual health research with young adolescents, highlighting its potential benefits to provide valuable insights into their sexual development and the support that they need, as well as shaping their knowledge before they are exposed to incorrect information. One participant said,

*“I think that engaging young adolescents in research would be very helpful, because now we’ll be able to see where their minds are at, what they do know or what they have experienced. And then that allows us to build on what they have provided us with when we are thinking of their care* [meaning sexual health support and intervention]*”*

(staff from the youth recreation center)

The narratives also revealed that young adolescents are already exposed to conversations and messages about sex through social media, school, their peers, and the world around them, making it critical to engage them early with accurate, affirming, and developmentally appropriate sexual health education. A youth counselor participant said,

*“So, my views are that they’re probably seeing it, It’s everywhere, right? Social media, their friends are probably talking about it. So, I think it’s important to make sure to reach them young because they’re going to hear about it and others are already hearing it. And if I would, I would rather have a youth have a positive outlook on safe sex than a negative outlook. Because 5, 10, 15 years down the line they may have an STD or an STI or have a child which may impact their self-esteem. So maybe if someone can sit down with them: well, what are your views on sexual health? Then, why do you feel this way? That can help”.*

Another participant added,

*“Because it’s really important to gauge their knowledge while they are young. If we think back to when we were between 10 and 14, with all the misinformation, I believe that it may still be the case, that kids talk, they have misinformation. But we don’t know what is it that they’re believing nowadays? But kids, need to know what’s going on around at this age. So, we need to know, what they know so we can give them more information, and one way of knowing is to engage the in research”*

(a sexual health education teacher)

#### 3.1.2. A Missing Piece

The fact that research among young adolescents is missing was another theme that emerged from the participants’ narratives. The findings showed that research with young adolescents is viewed as important, yet it is a largely unexplored area that could significantly enhance sexual health support and education strategies. A social worker working with children with disabilities said,

*“Hearing sexual health issues from the perspective of a young person, I absolutely think that’s a missing piece and an incredibly valuable area that I would imagine has gone largely unexplored. Not to say exclusively, but what would that be like to actually work with young people and hearing from them what has been helpful, what has been missing in terms of them better understanding their bodies, their development, attitudes towards sexual health and development, the supports that they need What are the barriers to that? Yeah, that would be a very different conversation, I think that would be a much-needed perspective to have.”*

#### 3.1.3. Safety as a Pathway to Young Adolescent Engagement in Research

Ensuring safety among young adolescents participating in research also emerged as another element critical when engaging them in sexual health research. Participants reported that young adolescents are more likely to open up and respond to questions related to sexual health as long as they are assured of confidentiality. A member of a youth-based organization said,

*“I’m just going to be blunt. 10- to 14-year-olds really like to talk. Sometimes it’s unhealthy chatter. What we need is to make sure we provide that safe space for them so that they can talk. I will say you should build in a confidentiality. Tell them that, “we want you to allow us talk to you in this space. This is the forum and platform for that space. However, though, if we do hear something, then that is going to lead to more questioning and making sure we are protecting you and your safety.”*

The participants also reported how open young adolescents are to sharing their experiences when given a safe space. A health education teacher also said, *“I think that younger people are just more open and honest about these conversations than they were before”.* Similarly, a social worker commented, *“Well, again, as I said, I’m always amazed with these young people. When you create a safe, therapeutic and confidential setting, they almost will say anything”.*

In support of the feasibility of conducting sexual health research with young adolescents and the critical nature of ensuring safety and confidentiality when engaging young adolescents in sexual health research, the majority of the participants referenced their experiences of having sexual-related conversations with this age group. One participant stated,

*“I mean, I ask them straight out. They’re always like, ‘Miss, that’s so uncomfortable,’ but I’m like, ‘What does sex mean to you,’ and they’re like, ‘It’s the first time we’ve met with you,’ and I’m like, ‘I know, this is the first time you meet with me, so uncomfortable, but this is our safe space.’ I try to make a joke out of it. They’ll be like, ‘Well, it means two people’s private parts are touching.’ ‘Great. What do you do to make sure that that’s safe?’ So, it depends on the youth and how you convince them of their safety”*

(a youth counselor)

### 3.2. Recruitment Strategies

The participants’ narratives revealed different strategies for recruiting young adolescents into sexual health research. Four notable strategies were evident.

#### 3.2.1. Parental Understanding as a Gateway to Recruitment

Narratives from all participants revealed parental awareness and an understanding of the study as the most crucial recruitment strategy. The findings showed that parents could decline participation simply due to limited knowledge or a lack of understanding of young adolescents’ sexual health. One participant shared,

*“You need parents and guardians. You need their buy-in, because a lot of times, they get scared. ‘What are you talking to my kids about?’ And there’s this whole idea, like, that’s* [meaning having sexual health conversations] *not someone’s responsibility or the school’s responsibility, that’s the parents’ responsibility. So, unless they understand what you are going to talk about and why, they cannot allow their kids to participate. I would actually say, you come with evidence and what exactly you need them [the young adolescents] to do. Those things will help them understand. Because sometimes if you say sexual, and they are like, ‘oh no, you’re not talking to my child about this and that.’ So, use terms that they would understand, let them know how it’s going to impact them as parents, how it’s going to impact their child and what’s going to happen”*

(staff at a recreation center)

The findings also revealed that some parents lack education about sexual health development for young adolescents, so, in addition to being an important recruitment strategy, collaborating with them when engaging their children in sexual health research provides an opportunity to educate and empower them with the knowledge and tools to have open conversations with their children. A health education teacher commented,

*“I really think doing more targeted work with parents. Because that’s a big barrier. I think because a lot of parents today likely didn’t receive good enough sexual health education. So, engaging them in research provides an opportunity to give them information. Maybe if someone can sit down with them [parents], well, what are your views on sexual health then? Why do you feel this way? And then introduce your research and them facts about the importance of the research, it can help. I think when a parent feels heard and seen, then they’re more likely to hear and see their own children with sexual health lens and be open to allow them to participate. And I think once you start with the parent and getting their information, their views, and then talk with the child on their own, it can help to have a more balanced views and a well-informed approach to supporting young adolescents”*

(a youth counselor)

#### 3.2.2. Collaboration with Schools and Youth Organizations

The insights gathered from the participants emphasized the importance of leveraging school connections and community groups to effectively engage young adolescents in conversations about sexual health. The findings highlighted partnering with community organizations that are already working with schools, as such partnerships can enhance trust, improve access to young adolescents, and align research efforts with existing educational initiatives. One participant said,

*“I have found that it’s easier when you have a relationship with the school and then the school does some of that legwork because it means more coming from the school than from the researcher. Talking to the community engagement person, community organizations already working with schools, or the assistant principal and explain, ‘This is what we’re doing, could you reach out to parents for us?’ And then flyers on social media and just places that parents and youth might engage with”*

(member of a youth-based organization)

Another participant said,

*“I feel like the best connection is within the schools because the kids are there, especially a lot of times after school, like pick up and stuff like that. Also, a lot of the schools will have parent liaisons or connection with the parents that way”*

(a youth counselor)

#### 3.2.3. Providing a Safe Space

Creating a safe, confidential, and non-judgmental environment also emerged as an important strategy for recruiting young adolescents in sexual health research. The findings showed that, by providing a supportive atmosphere and listening without judgment, young adolescents can be motivated to participate and be open to engaging in conversations. A youth counselor said,

*“I would definitely say building a relationship with the youth where they know that you are a safe place and that you’re not going to run back and tell mom and dad what they said, or you’re not going to run and go tell someone else. And just making sure that they are reassured that their information stays confidential.”*

The findings also underscored the importance of balancing young adolescents’ autonomy with ensuring their safety, including helping them to understand situations where confidentiality may need to be broken to protect them. As one member of the youth organization explained,

*“I will say you should build in confidentiality. Tell them that: ‘We want you to be safe. This is the forum and platform for that safe space. However, though, if we do hear something, then that is going to lead to more questioning and making sure we are protecting you and your safety.’”*

#### 3.2.4. Utilizing Non-Sexualized Messaging

The findings highlighted the importance of using non-sexualized messaging as a critical strategy for recruiting young adolescents into sexual health research. Community stakeholders emphasized that, while young adolescents may be more open to participating than their parents, recruitment efforts should avoid framing the study explicitly around sexuality. As one adolescent clinic health provider noted,

*“I feel like the kids would be more inclined to join than the parents. I don’t know. Would you call it* [the study] *sexuality? That is my question. I don’t know. I have an eight-year-old. He’s almost nine in a couple months. If I asked him that, he would be like, ugh. He would be like mortified and embarrassed, I think I just wonder if you could call it something else that’s a little less intimidating to a kid that age, that might be something to think about.”*

#### 3.2.5. Incentives

Participants’ narratives also revealed incentives as another important recruitment strategy for young adolescents. Money and other forms of incentives were given as examples:

*“I would say it’s definitely hard to recruit youth, but I would say incentives, sometimes food is a really big incentive. If you come here, there will be pizza or maybe there’ll be insomnia cookies. Or if you participate, there is a $10 gift card, or $20 gift card. So, I think incentives is a big one”*

(a youth counselor)

Another youth counselor participant similarly stated,

*“Being able to give them incentive in some way, not necessarily monetary incentive, but some type of incentive that would keep them wanting to participate. I don’t know. Something that’ll make them feel appreciated.”*

### 3.3. Research Questions

The findings also revealed some sexual health-related questions that researchers could ask young adolescents. These questions centered around sexual health knowledge, sexual health education, perceptions of other people, sexual health needs, and sources of sexual health knowledge. Many participants suggested starting with general inquiries about adolescents’ existing knowledge of sex and STIs. Table 2 provides a list of the participants’ reported questions and excerpts.

### 3.4. Buidling Readiness for Participation in Sexual Health Research

The findings also revealed the importance of introducing sexual health conversations early to normalize the topic and prepare young adolescents for meaningful engagement in research. Many participants reported that, when these conversations are developmentally appropriate and ongoing, they promote comfort, trust, and openness—critical foundations for effective participation in research. The findings also showed that exposure to early sexual health conversations can equip young adolescents with the language and confidence to articulate their experiences and reduce discomfort and stigma around sexual health topics. One participant, a youth counselor, reported that *“starting these discussions* [sexual health conversations] *early makes the conversation less awkward later when they are engaged in research and helps them respond to questions clearly.”* This readiness was seen as essential in ensuring that young adolescents can actively and confidently contribute to research that reflects their needs and perspectives.

Understanding the appropriate age at which to begin sexual health conversations emerged as a critical factor in preparing young adolescents for participation in sexual health research, as well as in cases of sexual abuse. Although the suggested starting ages ranged from toddlerhood to age 12, the findings consistently emphasized the value of early engagement in order to build comfort and familiarity with these topics, establishing a strong foundation for future research participation. One participant said,

*“It really starts when they* [children] *are getting curious about their private parts. We really need to teach our children the proper names of our body parts. And so that’s a sexual health conversation. I’ve had conversation with my adult children, that, ‘Okay, we’re not using these cute names, you need to tell your son, ‘That is a penis.’ You need to tell your daughters that they have a vagina, so they can say, ‘he had me touch his penis’ or ‘he put his finger on my vagina’. So really, sexual health incorporates that at a very young age. We’re really talking probably at 18 months to two years when they’re starting to talk and recognize those things. When we normalize these conversations, they cannot feel embarrassed to talk about them in a research setting”*

(a health educator)

A participant who advocated for ages 5 to 6 said,

*“I think five is appropriate. I think five-year-olds are very touchy with each other. They are still exploring their bodies. And I think learning about consent at that age is developmentally appropriate. I think they’ve already learned about sharing. Right? That’s developmentally appropriate. And so, sharing their bodies with other people, that conversation makes sense at that. I think sex education that young adolescents should look like relationship building, friendship building, and what it’s like to listen to your body. Starting early like this lays the groundwork and helps prepare them to meaningfully engage in sexual health conversations—and, eventually, in research”*

(a health counselor)

The findings also suggested the need to delay initiating sexual health conversations until children are older to ensure that they can fully comprehend the information. Nevertheless, there was a consensus around the importance of considering the child’s developmental age rather than just their chronological age. An adolescent clinic provider shared,

*“I think somewhere between eight and 10, depending on the kid’s developmental stage. My son is eight right now and I just bought a book, a children’s book to review with him because I did not really feel ready and prepared to talk with him about it, but I heard that one of his little peers at school was an eight-year-old and making little like jokes and comments. And I was like, oh, okay, well he’s hearing it at school. I want to make sure that I can chat with them at home about some things at high level.”*

A health educator also added,

*“I would say I think 12 is a good age to start engaging them in sexual health conversations and is a good time too that they can participate in research, but I can also, say that I think we’re living in a different time, in a different generation. And so maybe there may be a need to go younger and I think it’s important to use language that is appropriate for each age group, right? So, the language you may use with a 16, 17-year-old, you probably shouldn’t use with a 12-year-old.”*

## 4. Discussion

This study examined community stakeholders’ perspectives on engaging young adolescents in sexual health research, seeking to understand how to engage this age group in research that could inform effective, age-appropriate sexual health development support interventions. We identified four major themes: perspectives on conducting sexual health research with young adolescents, recruitment strategies, research questions appropriate for young adolescents, and the perceived age for initiating sexual health conversations. Overall, the findings indicate that, although conducting sexual health research with young adolescents poses certain challenges, it is both feasible and necessary. The strong support expressed by the community stakeholders suggests the urgent need to actively involve young adolescents in the design and development of sexual health research. This support also presents an opportunity for researchers to shift from a traditionally older adolescent- and adult-centered approach to one that meaningfully includes young adolescents, ensuring that interventions are responsive to their specific developmental needs, lived experiences, and voices.

Consistent with previous studies [3,6], this study found that the involvement of young adolescents in sexual health research remains a critical gap. This is particularly concerning in light of prior targeted efforts aimed at increasing the inclusion of young adolescents in such research [14,19]. Although progress has been made in advancing the engagement of youth more broadly in sexual health research [62], the current guidelines for involving young adolescents remain narrow in scope, limited in content, and often lacking in contextual relevance, thereby constraining the development of effective, age-appropriate sexual health interventions [63]. These findings underscore the need for effective strategies to facilitate young adolescents’ active and meaningful participation in research. Wilson et al. [62] proposed several strategies for enhancing youth involvement in research, including the development of best practices for youth engagement; providing training for researchers, young people, and stakeholders on these best practices; building new or expanding existing networks of researchers and other organizations focused on involving young people in health research; strengthening and standardizing the monitoring and evaluation of young people’s involvement in research; and increasing funding dedicated to youth-partnered research. However, further research is needed to evaluate the implementation and impacts of these recommendations specifically in the context of sexual health research with 10–14-year-old young adolescents. There is also a pressing need to explore innovative models of youth engagement and to examine the structural and ethical barriers that may hinder their meaningful participation. Importantly, research should prioritize understanding how young adolescents themselves wish to be involved in the research process, ensuring that their voices shape not only the content but also the methods and ethics of future sexual health studies.

The finding that researchers investigating young adolescents’ sexual health development support should engage parents is critical in advancing young adolescent sexual health research. In most parts of the world, parents and caregivers have the final say in determining the participation of their children in research, and they also play a crucial role in shaping their children’s attitudes and behaviors regarding sexual health. However, the findings of this study revealed that many parents feel unequipped to discuss sexual health with their children due to discomfort and a lack of knowledge, which may also affect their willingness to provide consent for young adolescents to participate in sexual health research. This aligns with previous research indicating that many parents are uncomfortable engaging in sexual health conversations with their children and that parental discomfort exists regarding sexual health discussions [64,65], suggesting the need for interventions that empower parents with sexual health information and the skills to handle such conversations with their children.

The challenges of obtaining parental consent, particularly in sexual health research, have been reported in previous studies [21,62]. However, unlike what has been suggested in previous research on the need to waive parental consent [66] as it limits participation and can bias samples toward adolescents who are comfortable discussing sensitive issues with their parents [1,10,31,32,33], the participants in this study suggested the need to collaborate with parents in sexual health research and enhance their literacy in young adolescent sexual health research through culturally responsive education and resources. This is consistent with Aventin et al. [65], who also found that providing accessible workshops, community discussions, and digital resources could bridge knowledge gaps and reduce discomfort. Additionally, early parental involvement in the research process through clear communication about the study’s purpose, benefits, confidentiality, and protections could offer greater support and reduce the resistance to young adolescents’ participation in sexual health research. Nevertheless, future research should explore parents’ perspectives on engaging young adolescents in sexual health research. In doing so, researchers can gain a more comprehensive understanding of the broader range of parental attitudes and barriers and determine strategies by which to navigate the issue of obtaining parental consent for young adolescents to participate in sexual health research.

This study also identified effective strategies for recruiting young adolescents to participate in sexual health research. The recommendation to leverage school connections has also been reported in previous studies, which have also described schools as trusted institutions within the community [67]. Utilizing peer networks, social media, community organizations, and school partnerships, as emphasized by the participants in this study, has also been found to be critical in the research process and reported to make research more accessible and appealing [68]. By leveraging these youth-centered outreach methods, researchers can reach a broader and more diverse group of participants while maintaining ethical recruitment practices [69]. Additionally, building strong relationships with key stakeholders, such as parents, educators, and community organizations, not only reinforces the legitimacy of the research but also helps to create a supportive environment in which adolescents feel safe to participate [24,68]. However, these relationships must be balanced with a clear commitment to protecting young adolescents’ confidentiality, a factor that is crucial in promoting their trust and encouraging honest, open engagement in sexual health research.

The use of incentives also emerged as an important component in the recruitment of young adolescents in sexual health research. While monetary incentives were mentioned, other forms of incentives, such as providing leadership opportunities or creating roles within the research process, like involving them in a youth advisory board, were also suggested as effective means of engaging young adolescents in sexual health research. This finding aligns with previous studies [21,38] that suggest that non-monetary incentives can promote a sense of ownership and responsibility among adolescents, which can encourage active participation.

The findings of this study also showed that the successful engagement of young adolescents in sexual health research requires establishing trust with them. This is consistent with Crane and Broome’s [24] finding that trust is a significant factor in young people’s willingness to participate in research. Therefore, to successfully engage young adolescents in sexual health research, it is essential to prioritize trust-building strategies that facilitate a sense of safety, transparency, and collaboration. This not only enhances their comfort but also encourages meaningful participation.

Ethical and transparent research practices also play a critical role in building trust. As the community stakeholders emphasized in this study, researchers must communicate the study objectives, procedures, and potential benefits to young participants and their guardians to promote informed decision-making and voluntary participation. This is consistent with previous research [70] that underscores the importance of fully disclosing study information, including the right to decline participation without consequence, as a key component in respecting participants’ autonomy. In addition, ensuring that research settings are free from judgment and coercion allows young participants to share their perspectives confidently and authentically [71].

The findings also revealed some questions that are appropriate for young adolescents when engaging them in sexual health research. Knowledge of which questions are appropriate for young adolescents is crucial because this is a group that has been sidelined in sexual health research and intervention development, in part due to the challenges of formulating developmentally appropriate sexual health questions [17]. Research indicates that thoughtful, age-appropriate questioning not only enhances participant engagement but also encourages self-reflection, which can contribute to improved sexual health outcomes [72]. Future research should focus on testing the appropriateness of the proposed questions among young adolescents and exploring how young adolescents understand, interpret, and respond to them. Additionally, co-creating research questions with young adolescents can help to ensure that sexual health questions are developmentally appropriate, relevant, and grounded in their lived experiences. Engaging young adolescents in this way not only improves the clarity and appropriateness of the questions but also elevates the issues that they identify as most important.

Introducing sexual health conversations early was identified as a key factor in engaging young adolescents in sexual health research as it was viewed as laying the foundation for their participation in sexual health research. Participants expressed varied perspectives on the appropriate age for initiating these conversations, with many emphasizing the value of early exposure to concepts such as consent, bodily autonomy, and personal boundaries. Participants perceived these conversations as a way to normalize these often stigmatized topics in sexual health and prepare young adolescents for open discussions in research settings. Evidence suggests embedding sexual health conversations in early childhood development to help young people in building the knowledge, language, and confidence needed to navigate their health and relationships [73,74]. As a result, by the time that they are invited to participate in research, they are more developmentally equipped to understand the research questions and articulate their experiences. These findings point to the need for integrated, age-appropriate sexual health education that begins in early childhood and is reinforced across developmental stages. To inform and guide these efforts, future longitudinal research is needed to determine how the early introduction of sexual health education influences young adolescents’ cognitive and emotional readiness to participate in sexual health research. Such evidence is essential to support the design of ethical, effective, and developmentally aligned sexual health research and interventions.

The finding that sexual health conversations should begin as early as toddlerhood reinforces the long-standing recommendation that educational policies should mandate comprehensive, age-appropriate sexual health education that begins in pre-school and progresses throughout the child’s development [75,76,77]. The United Nations’ technical guidance recommends that sexual health education should be integrated into school curricula from age 5, gradually building on foundational topics that are scientifically accurate and tailored to different ages [75,76]. Parents and caregivers must also be provided with the necessary resources and support to facilitate these conversations at home, ensuring that learning extends beyond the classroom [75,78]. Additionally, educators should receive specialized training to deliver this content in ways that align with students’ cognitive and emotional growth. Cultural and community considerations must also be integrated into these efforts, ensuring that sexual health education is inclusive and reflective of diverse values and beliefs [79]. Therefore, further research is needed to identify effective strategies to enable parents and caregivers to initiate early and developmentally appropriate sexual health conversations with their children. Additionally, longitudinal studies are needed to explore how early parental involvement in sexual health education influences young adolescents’ readiness to engage in sexual health research and impacts their long-term sexual health outcomes.

### 4.1. Implications

This study has implications for research, practice, and education. The strong community readiness to conduct research on this topic, as evidenced by the support from community stakeholders, presents a significant opportunity for researchers to design studies that center young adolescents’ perspectives.

This study also highlights the pressing need for educational efforts targeting parents and caregivers’ empowerment to engage young adolescents in sexual health conversations. Parents determine whether young adolescents participate in research or not and play a pivotal role in shaping children and adolescents’ attitudes and sexual health behaviors. However, this study revealed that many lack the knowledge or confidence to have open and informed discussions with their children. The findings of this study may inform interventions that equip parents with accurate information about research and sexual health that can help them to become more open to allowing their children to participate in sexual health research.

The study underscores the importance of strategic recruitment and collaboration with schools and community organizations when conducting sexual health research with young adolescents. Establishing strong partnerships with these institutions can enhance multidisciplinary involvement and ensure that research efforts are well received and impactful.

This study also has important implications for the implementation of sexual health research and comprehensive sexual health education for young adolescents. The findings underscore the critical need to engage young adolescents in ways that are responsive to their developmental stages, cultural contexts, and lived experiences. By centering young adolescent voices and perspectives, researchers and health educators can design research methodologies and educational interventions that are not only age-appropriate but also culturally relevant and grounded in the everyday realities of this population. Such an approach increases the likelihood of meaningful engagement, knowledge retention, and behavioral impact.

### 4.2. Limitations

This study was context-specific; thus, the findings apply to Western New York. The social and cultural factors influencing attitudes toward sexual health research for young adolescents vary widely across different regions and communities. What is perceived as a facilitator or barrier in one setting may not hold in another, limiting the transferability of the study’s conclusions. Moreover, discussions about sexual health research involving young adolescents can be influenced by ethical concerns, cultural taboos, and discomfort, which may shape the depth and openness of stakeholder responses. Additionally, the study was limited to the perspectives of community stakeholders who were professionals with substantial experience in adolescent sexual health and youth development. While their expertise enriched the findings, it may have also influenced their responses, as they were likely to draw on their professional training and frameworks. Perspectives from individuals without formal training or professional roles, such as parents, caregivers, or community members with lived experience, may offer different insights. Future research should also incorporate the voices of untrained community members, young adolescents, and their caregivers to provide a more comprehensive understanding. The sample was also predominantly female, which may have limited the range of perspectives captured, particularly those of male or gender-diverse stakeholders. This gender imbalance likely reflects the demographics of those most engaged in youth sexual health work in our study setting. Future research should aim to include a more diverse range of participants to broaden the understanding of community-level support for young adolescents’ sexual health development. Moreover, this paper does not provide details of the methodological approach that can be used to engage young adolescents, as this work was addressed in phase four of the main study and will be reported in a separate paper. As such, the current research was limited in its ability to fully describe the processes related to young adolescent study design.

## 5. Conclusions

Engaging young adolescents in sexual health research is essential in informing developmentally appropriate and equitable interventions. However, as this study highlights, the successful recruitment of this age group in sexual health research requires a comprehensive, multifaceted approach that prioritizes trust building, ethical transparency, and meaningful collaboration with parents, schools, and community stakeholders. The findings showed that trust, particularly among researchers and young adolescents and their families, is foundational to young adolescents’ willingness to participate in sexual health research. The findings also underscored the importance of culturally and contextually responsive strategies tailored to the needs and concerns of the communities being served. These findings reinforce the critical role that community stakeholders play in facilitating sexual health research access to young adolescents and shaping the conditions under which participation can occur. Future studies should build on these insights to further refine recruitment strategies and ensure that young adolescents’ voices are represented authentically, safely, and ethically in sexual health research. By centering stakeholder perspectives, researchers can create more inclusive and supportive environments that advance sexual health equity for young adolescents.

## Figures and Tables

**Table 1:** Sociodemographic characteristics of the participants.

Demographic Characteristics		Range/*M*	*n*	*%*
Age (years)		23–59/41		
Sex	Female		16	94.1
	Male		1	5.9
Race	White		7	41.2
	Black/African American		9	52.9
	Mixed race: Caucasian–Indian American		1	5.9
Education	Undergraduate		7	41.2
	Master’s degree		10	58.8
Youth-Related Role	Members of youth-based community organizations		4	23.5
	Health education teachers		4	23.5
	Youth counselors		4	23.5
	Adolescent healthcare providers		2	11.8
	Youth recreation center staff		1	5.9
	Staff working with youth with disabilities		2	11.8

**Table 2:** Sexual health questions appropriate for young adolescents and excerpts.

Question Area	Question Category	Excerpt
Sexual health knowledge	What young adolescents know about sex	“Some of the questions would be, ‘What do you know about sex?’ Find out what they know. And if you start there, maybe you can build on it. What is it that they know? Questions like, ‘Can you get pregnant the first time you have sex?’” (a member of a youth organization board).
		“It depends on the child. If we have an 11-year-old kid come in and he has a history of maybe gang violence or things like that, I am going to jump right to our questions and expect him to understand. If I have a kid come in who appears to be super innocent, I’m going to start with, ‘What does sex mean to you?’ So, it’s really just understanding your population” (a youth counselor).
	Age at which they were exposed to sexual health information	“What to ask them? I mean, just when did they become knowledgeable about certain sexual health topics? Are they knowledgeable? Have they heard of what an STD is or how they’re transmitted? I just think a female’s cycle, her menstrual cycle, is important. I mean, some of our females are getting their cycle at school for the first time and have no idea what it is and are almost in some sort of hysteria because they’re just confused. So that shouldn’t happen to any young girl” (a member of a youth-based organization).
	Knowledge of STIs and menstrual cycle	“I think asking those questions of, well, how do you feel about sex? What do you feel about STDs? What do you feel about the transmission rate? Are you sexually active? Are you around people who are sexually active? What do your friends feel about sex or how they think about it?” (a youth counselor).
	Perceptions of having sexual health conversations	“I guess one question would be is are you open to having conversations about sexual health? Do you know what that means? What does that look like for you? How can we meet you where you are to provide what you need? How can we talk about sex and education in the way that you need? So, letting them help create things. What do you see as a need for yourself and for the community and for your peers?” (a member of a youth organization).
	Quality of support received during conversations	“I would imagine that asking them within the context of their own family of origin, have they felt that their questions and their curiosities have been adequately answered and supported as they are developing? Are they in an environment where they are able to really talk about questions that they have? And I think that’s a really important place to start because you absolutely can’t compare one young person’s experience to another” (a social worker).
Sexual education	Young adolescents’ perceptions of sexual health education	“Maybe what they think of the sex ed that they’ve received and whether or not it was too much or not enough or just right. I’ve had some young people who’ve been in our program for a couple years who said that we talk about too much sex stuff, but it’s a sexual health program. What do you mean? So, I’d be interested to see what their perceptions are and what their perception is of the education they get in school” (a member of a youth organization board).
Young adolescents’ perceptions of other people’s views on sexual health education	What young adolescents think other people think about sexuality education	“What they think their teachers think about that sexual education, and then just how comfortable they are talking to trusted adults about sexual health and then their peers” (a youth counselor).
Sexual health needs	What young adolescents’ sexual health needs are	“I guess just, what are their needs? Because I’m sure we’re not meeting all of their needs. So, I’d love to know what those needs are” (an adolescent healthcare provider).
Sources of sexual health knowledge	Where young adolescents obtain sexual health information	“It’s also important, to find out whether or not these young adolescents have siblings and what are their siblings sharing with them? Because that’s probably where they’re getting their information from. So probably I think if we had more information regarding that, we could probably do a better job in schools teaching sexual health. I think sexual health needs to be part of a curriculum so kids can make healthy choices” (a health education teacher).
		“It is important, to find out whether or not these young adolescents have siblings and what are their siblings sharing with them? Because that’s probably where they’re getting their information from” (a health education teacher).
	Exposure to peers	“What type of exposure do they have amongst young people? I have one student I work with who goes to a school of everyone who’s the same gender versus a co-educational setting. So that changes the dynamic of a person’s experience too” (a social worker).
	Young adolescents’ perceptions of the available resources	“Yeah. I think my biggest question would be, what resources are beneficial? Where are they getting information from? Are they getting it from social media? Are pamphlets even still beneficial? Do they use them? Do you know what pamphlets are and even look at them anymore? Where would be the best place for us, as adults, to reach them on these things? Because I know I’ve said over and over, we need more means of support. We need to get them resources, but I think it’s a conversation with them of what those resources look like and what they even need in the first place too” (an adolescent clinic healthcare provider).
	Sources of information	“I think questions for young adolescents include walking them through their social media about what they see in regard to sexual health, what they don’t see, what they experience around the music they listen to, their neighborhoods, talking to them less about what they’re learning in the classroom, and talk to them more about what they’re learning in life, will really help you navigate, ‘Okay, where do we fill in with education for them’” (a health education teacher).
Sexual behaviors	Dating experiences	“I think finding out if they’re knowledgeable about certain things or if they’ve already been dating. And if they say yes, I would feed questions off of those responses to dig a little deeper and cover the topics that are necessary and important” (a member of a youth-based organization).

## Data Availability

Data are not publicly available due to ethical restrictions but can be provided upon request from the corresponding author.

## Supplementary Materials

The following supporting information can be downloaded at: https://www.mdpi.com/article/10.3390/healthcare13141711/s1, Community Stakeholders’ Interview Guide.

## Abbreviations

The following abbreviations are used in this manuscript:

CBPR	Community-based participatory research
INSHHR	Interdisciplinary sexual health and HIV research
NY	New York
STIs	Sexually transmitted infections
SRH	Sexual and reproductive health
