# Supplementary material for: Community Stakeholders’ Perspectives on Recruiting Young Adolescents (Age 10–14) in Sexual Health Research
Source: Healthcare (Basel). 2025 Jul 16;13(14):1711. doi: 10.3390/healthcare13141711 (PMC12294775; doi:10.3390/healthcare13141711)
Supplement: Supplementary file 1 [file healthcare-13-01711-s001.zip › healthcare-3683503-supplementary.pdf]

Supplementary material

Community Stakeholders' Interview Guide

Instructions: Thank you for participating in our study. We appreciate your willingness to share your perspectives. We are interested in your thoughts about conducting sexual health research with young adolescents (ages 10-14). Our discussion may take up to 1 hour. As someone with valuable insights, your experience is essential, and we are grateful to learn from you.

We would like to have some conversations with young adolescents to understand their experiences with sexual health development support.

1. How do you feel about having conversations with young adolescents to learn more about what support they are getting when it comes to puberty, relationships, and making decisions about their sexual health?
   Probes:
   - What factors might facilitate engaging young adolescents in sexual health research?
   - What are the factors might make it difficult to engage young adolescents in sexual health research?

2. In your opinion, how best can we engage young adolescents in sexual health research and conversations?
   Probes:
   - What are the effective strategies for engaging adolescents aged 10–14 in discussions about their sexual health in a research setting?
   - What strategies have you seen work well, or would recommend for involving adolescents aged 10–14 in discussions about their sexual health in a research setting?
3. What kinds of questions do you think we should ask them that will help us to get information that will help us find better ways of supporting the young adolescents' sexual health development?
   Probes:
   - What specific questions could help us learn about their experiences with the support, guidance, and resources available to them?
   - Are there any topics we should avoid or approach with extra care?
